# Supplementary material for: Construction and integration of three de novo Japanese human genome assemblies toward a population-specific reference
Source: Nat Commun. 2021 Jan 11;12:226. doi: 10.1038/s41467-020-20146-8 (PMC7801658; doi:10.1038/s41467-020-20146-8)
Supplement: Supplementary file 1 — Supplementary Information [file 41467_2020_20146_MOESM1_ESM.pdf]

Supplementary Information

Construction and Integration of Three *De Novo* Japanese Human Genome Assemblies toward a Population-Specific Reference

Jun Takayama, Shu Tadaka, Kenji Yano, Fumiki Katsuoka, Chinatsu Gocho, Takamitsu Funayama, Satoshi Makino, Yasunobu Okamura, Atsuo Kikuchi, Sachiyo Sugimoto, Junko Kawashima, Akihito Otsuki, Mika Sakurai-Yageta, Jun Yasuda, Shigeo Kure, Kengo Kinoshita, Masayuki Yamamoto, and Gen Tamiya.

Supplementary Figure 1. Karyotypes of the three subjects.
Supplementary Figure 2. Inversions in chromosome 9.
Supplementary Figure 3. Workflow of the construction of JG1.
Supplementary Figure 4. Histogram of PacBio subread length.
Supplementary Figure 5. Histogram of Bionano optical map length.
Supplementary Figure 6. Circos plots showing the distribution of gap regions.
Supplementary Figure 7. Mate-pair sequencing.
Supplementary Figure 8. Majority decision.
Supplementary Figure 9. Gene prediction on JG1.
Supplementary Figure 10. PCA plots with PC1 or PC2 versus PC3.
Supplementary Figure 11. PCA plots with world-wide populations.
Supplementary Figure 12. PCA plots of "mock JG1" genomes.
Supplementary Figure 13. Length distributions of detected transposable elements in the GRCh38 and JG1 genomes.
Supplementary Figure 14. Mapping-based SV analysis.
Supplementary Figure 15. Alignment-based SV analysis.
Supplementary Figure 16. Genomic Sanger sequencing validation of the variants successfully identified by exome analysis using JG1.
Supplementary Figure 17. Comparison of the allele frequency between JG1 and hs37d5.

Supplementary Table 1. Basic statistics of PacBio subreads.
Supplementary Table 2. Basic statistics of Bionano optical maps.
Supplementary Table 3. Basic statistics of Illumina paired-end and mate-pair reads.
Supplementary Table 4. Assembly statistics for intermediate assemblies to construct JG1.
Supplementary Table 5. Basic statistics of Bionano assembly.
Supplementary Table 6. Comparison of basic assembly statistics with other high-quality assemblies.
Supplementary Table 7. Base-error rate estimation.
Supplementary Table 8. Length of consecutive Ns inserted manually.
Supplementary Table 9. Assembly evaluation and comparison.
Supplementary Table 10. Evaluation of AUGUSTUS gene prediction.
Supplementary Table 11. Alignment-based SNV calls supported by independent short- or long-read mapping experiments.
Supplementary Table 12. Basic statistics of DNBseq reads.
Supplementary Table 13. Basic statistics of nanopore long reads.
Supplementary Table 14. SV calls based on PacBio long-read mapping by NGMLR and SV calling by Sniffles
Supplementary Table 15. SV calls based on Nanopore super-long read mapping by minimap2 and SV calling by Sniffles
Supplementary Table 16. Alignment-based SV calls supported by orthogonal or independent mapping experiments
Supplementary Table 17. Sanger sequencing validation of exome-identified variants.
Supplementary Table 18. Effect of genic indel-specific correction of individual assembly.

Supplementary Methods. Manual correction of N-gaps length.

Supplementary Note 1. Majority decision and erroneous base inclusion.
Supplementary Note 2. Evaluation of *de novo* gene prediction.
Supplementary Note 3. JG1 haplotype location in PCA plot.

Supplementary References.

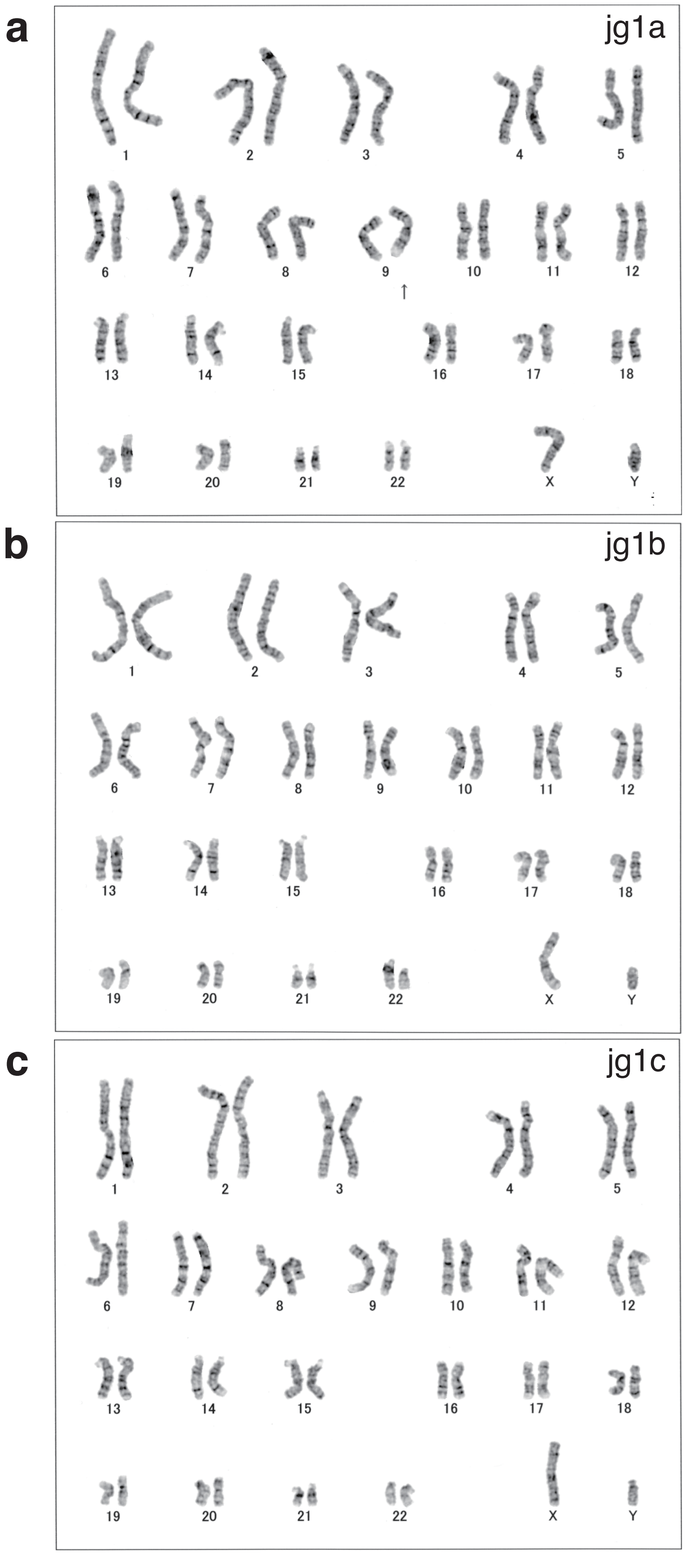

Supplementary Figure 1. Karyotypes of the three subjects. Shown are karyotypes for jg1a (**a**), jg1b (**b**), and jg1c (**c**). The arrow in **a** indicates the normal variation inv(9)(p12q13).

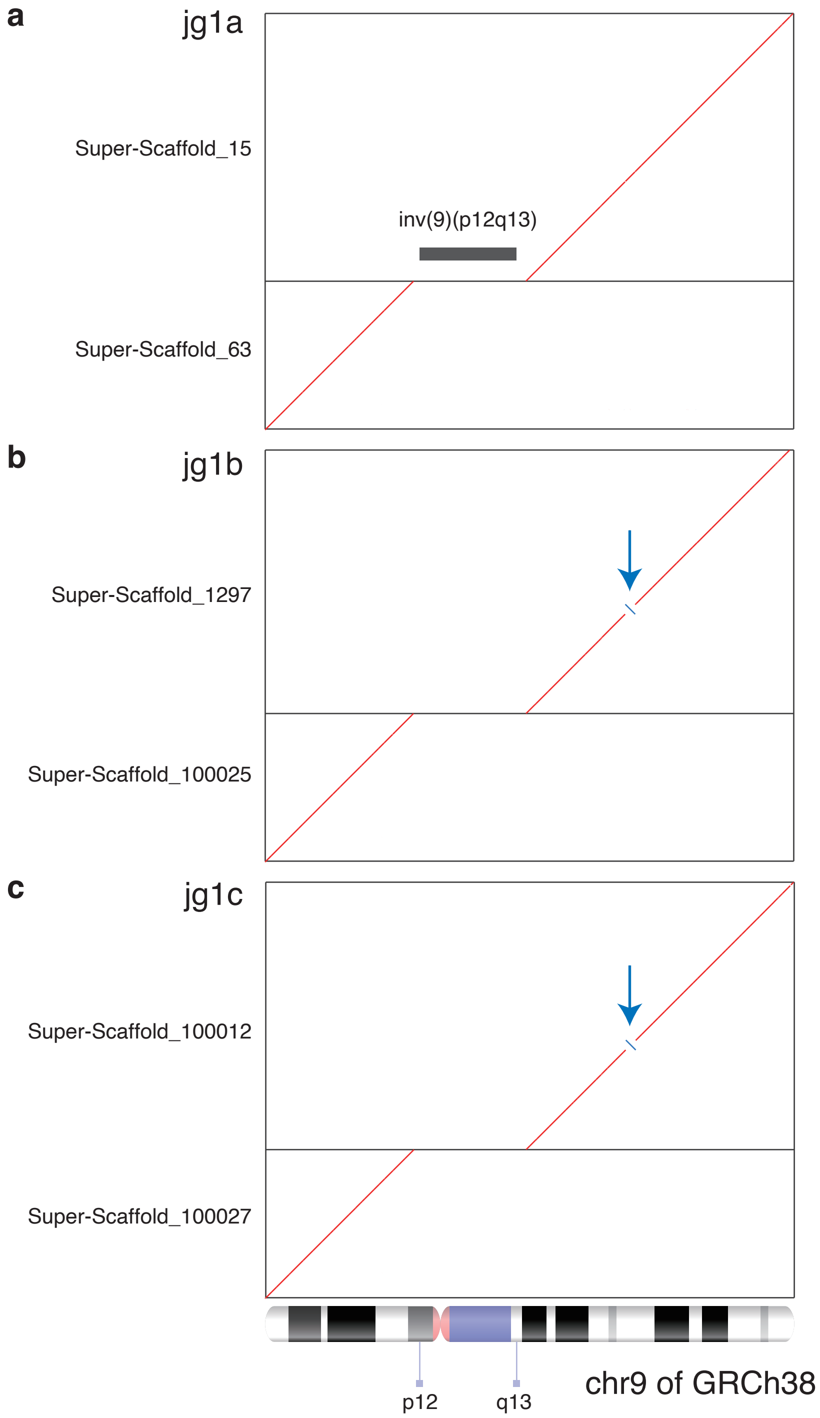

Supplementary Figure 2. Inversions in chromosome 9. Shown are Harr plots between chromosome 9 of GRCh38 and the two largest scaffolds aligned to chromosome 9 from the jg1a (**a**), jg1b (**b**), and jg1c (**c**) assemblies, indicating that the inv(9)(p12q13) in jg1a did not appear to affect the assembly and that the two individual genomes (**b** and **c**) harbor a possible shared inversion. 'Super-scaffold' is the default prefix designated by BionanoSolve software. The black bar in **a** indicates the position of inv(9)(p12q13). Blue arrows indicate the possible shared 2.6-Mb inversion.

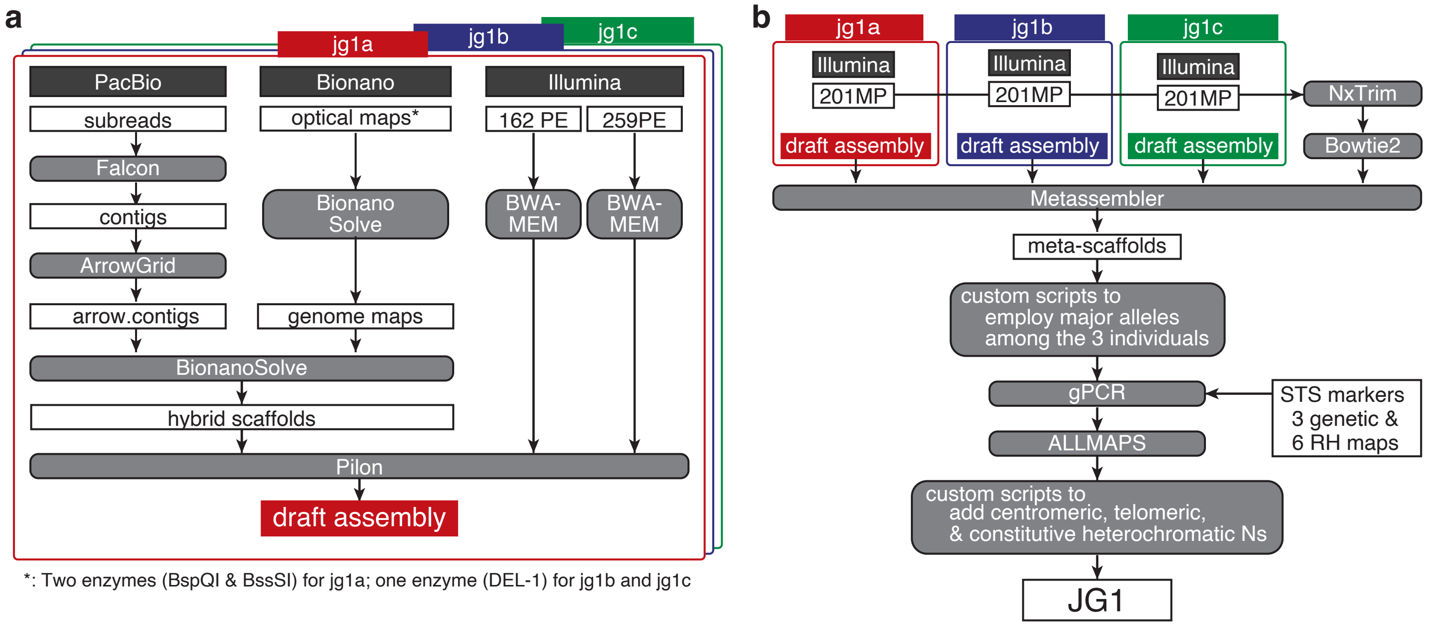

Supplementary Figure 3. Workflow of the construction of JG1. **a** Workflow of the construction of each draft assembly. **b** Workflow of the integration of the three draft assemblies. Rectangles indicate substrates such as reads, contigs, and scaffolds. Rectangles with rounded corners indicate software or processes.

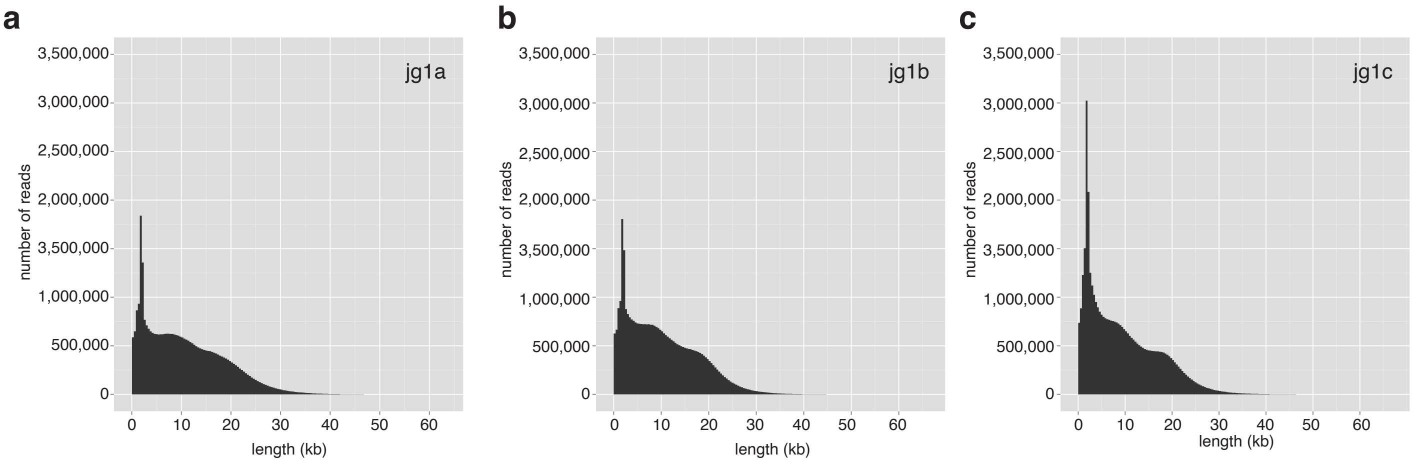

Supplementary Figure 4. Histogram of PacBio subread length. Shown are subread length histograms for jg1a (**a**), jg1b (**b**), and jg1c (**c**). The length of each subread was calculated using the SAMtools (ver. 1.8) faidx command.

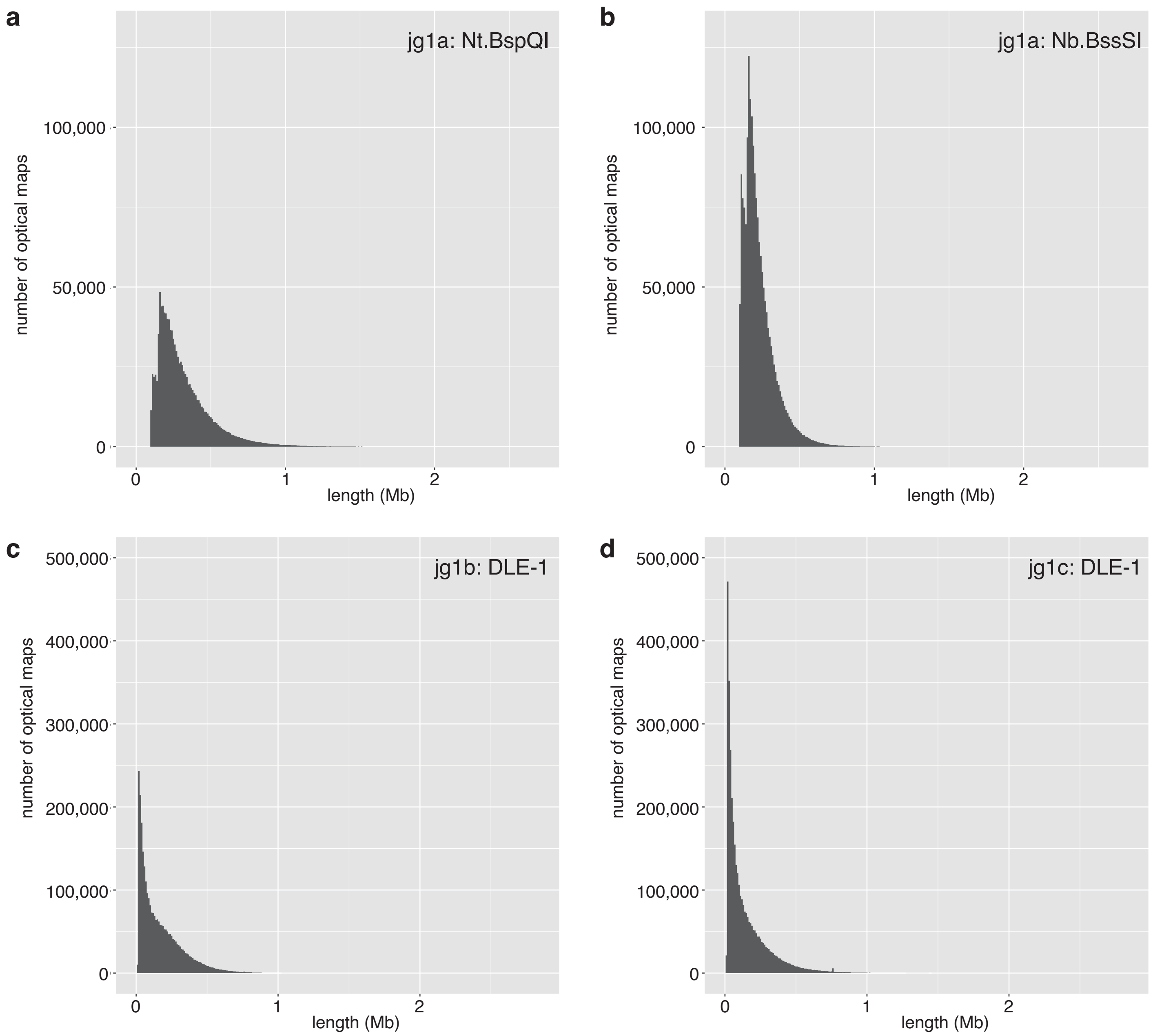

Supplementary Figure 5. Histogram of Bionano optical map length. Shown are histograms for Nt.BspQI (**a**) of jg1a, Nb.BssSI of jg1a (**b**), DLE-1 of jg1b (**c**), and DLE-1 of jg1c (**d**). The length of each optical map was extracted from the BNX file.

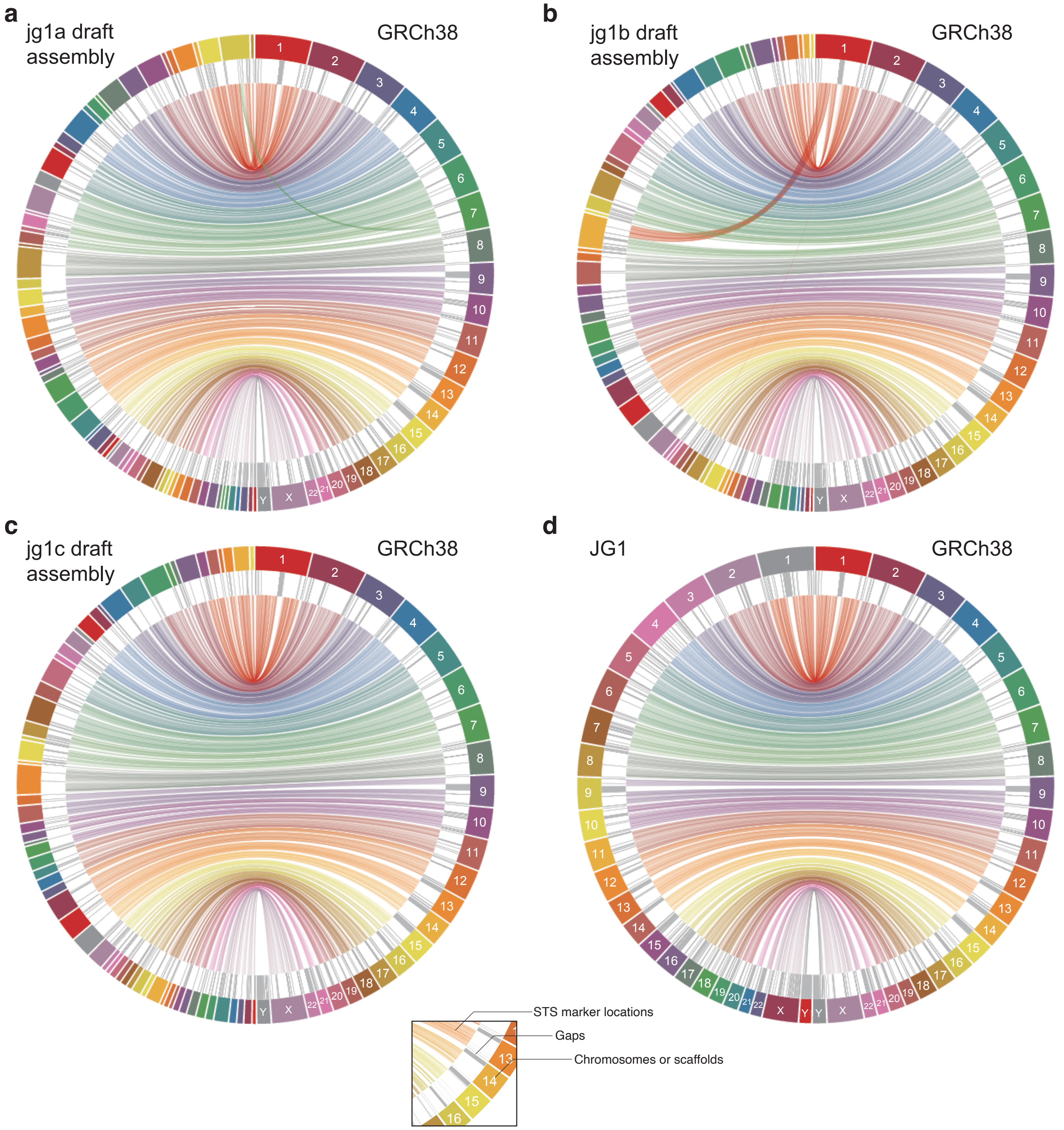

Supplementary Figure 6. Circos plots showing the distribution of gap regions. Shown are gap regions for individual draft assemblies (**a–c**) and JG1 (**d**) on the left side as well as the reference GRCh38 on the right side. The outermost track indicates the chromosomes, pseudo-molecules or scaffolds. The middle gray track indicates the distribution of gap regions. The links in the innermost track indicate the positions of STS markers from the deCODE genetic map for autosomes and the X chromosome and the Stanford-G3 RH map for the Y chromosome. Circos plots were drawn using Circa software (OMGenomics; www.omgenomics.com/circa).

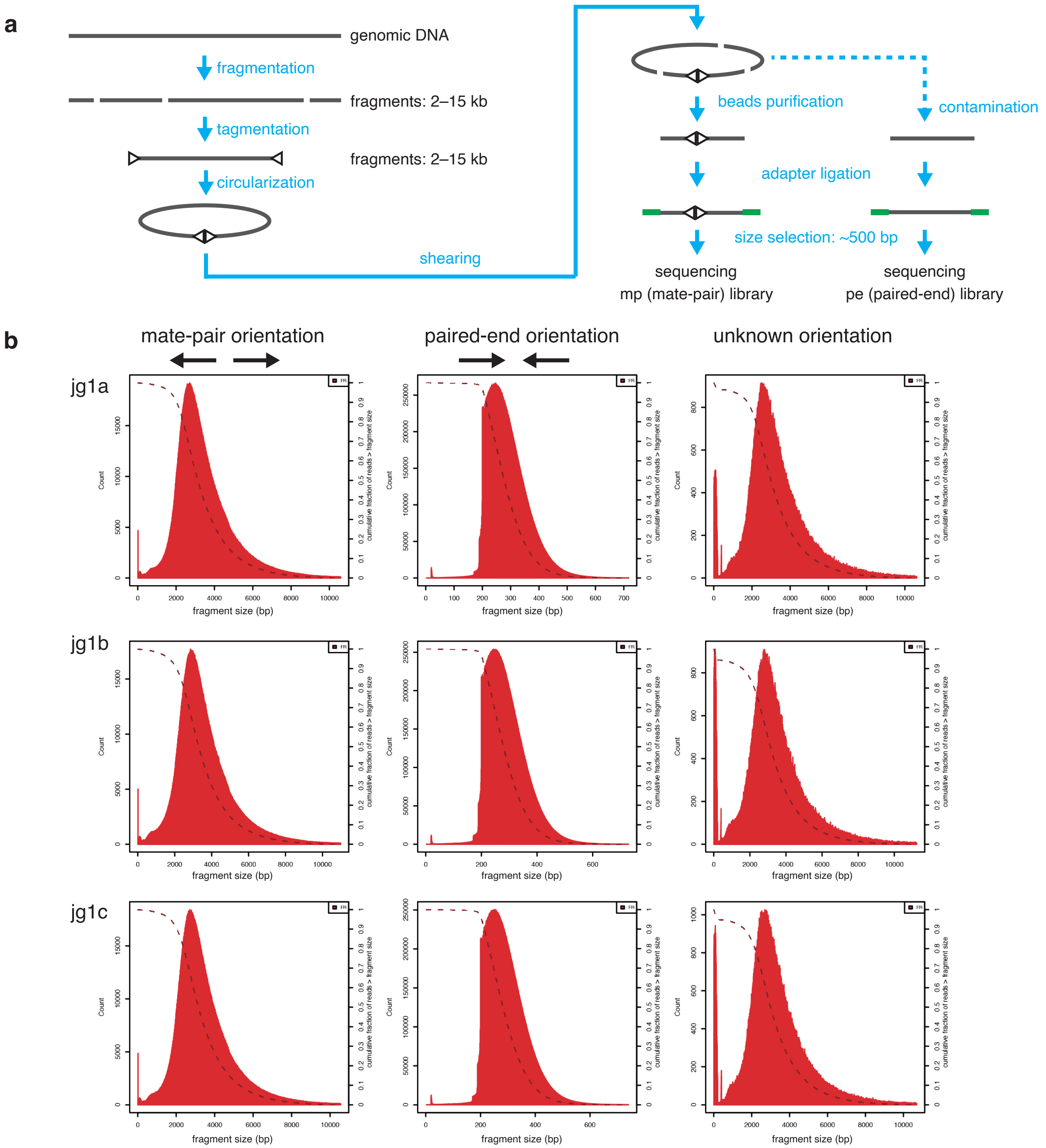

Supplementary Figure 7. Mate-pair sequencing. Procedure of mate-pair sequencing (**a**) and estimated fragment size distributions (**b**).

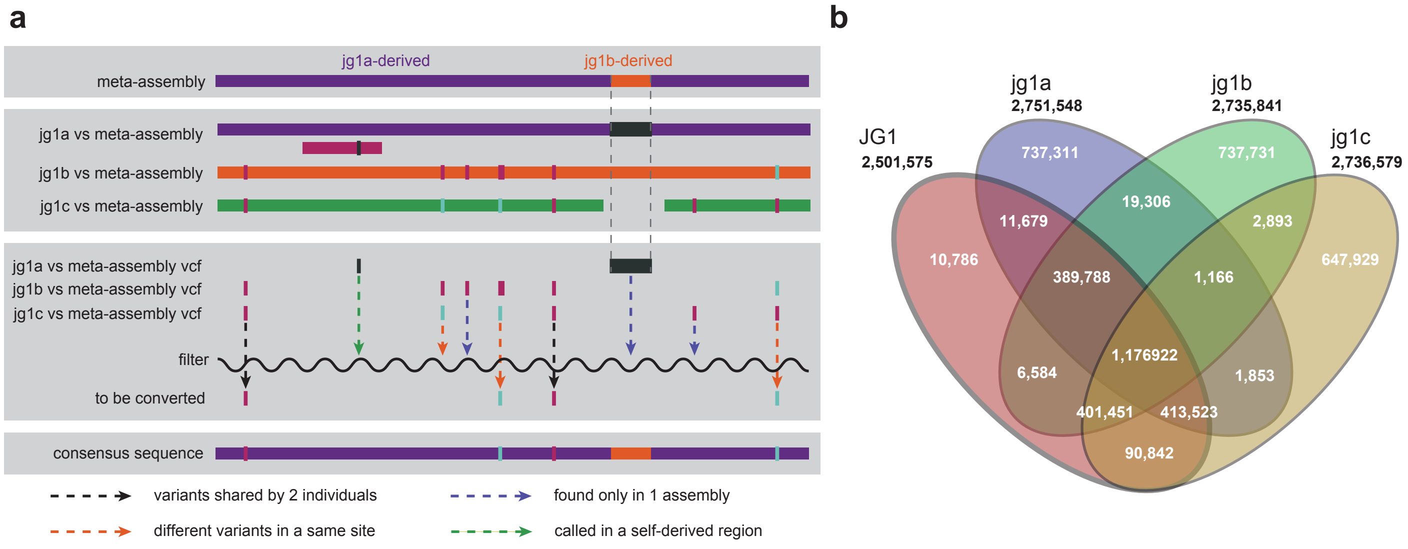

Supplementary Figure 8. Majority decision. **a** Schematic representation of the majority decision approach. **b** Venn diagram of SNVs detected in JG1, jg1a, jg1b, and jg1c by comparison with hs37d5. The intersection relationship was inferred using the BCFtools (ver. 1.8) isec command.

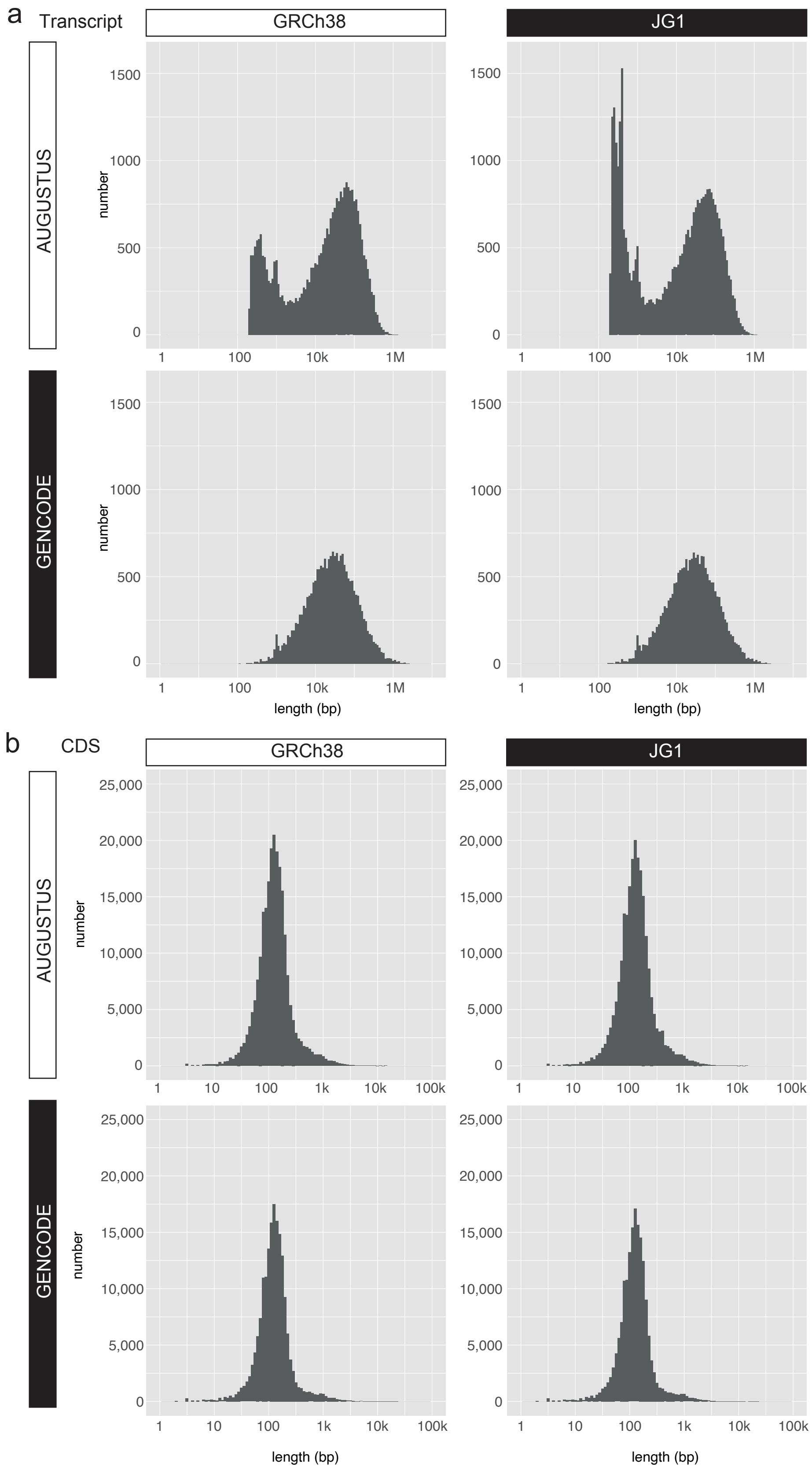

Supplementary Figure 9. Gene prediction on JG1. Length distribution of the predicted transcripts (**a**) or coding sequences (**b**) by AUGUSTUS on GRCh38 or JG1. Length distribution of the longest transcript per gene (**a**) or coding sequences (**b**) in GENCODE ver. 29 dataset on GRCh38 or those lifted over to JG1 is presented for comparison.

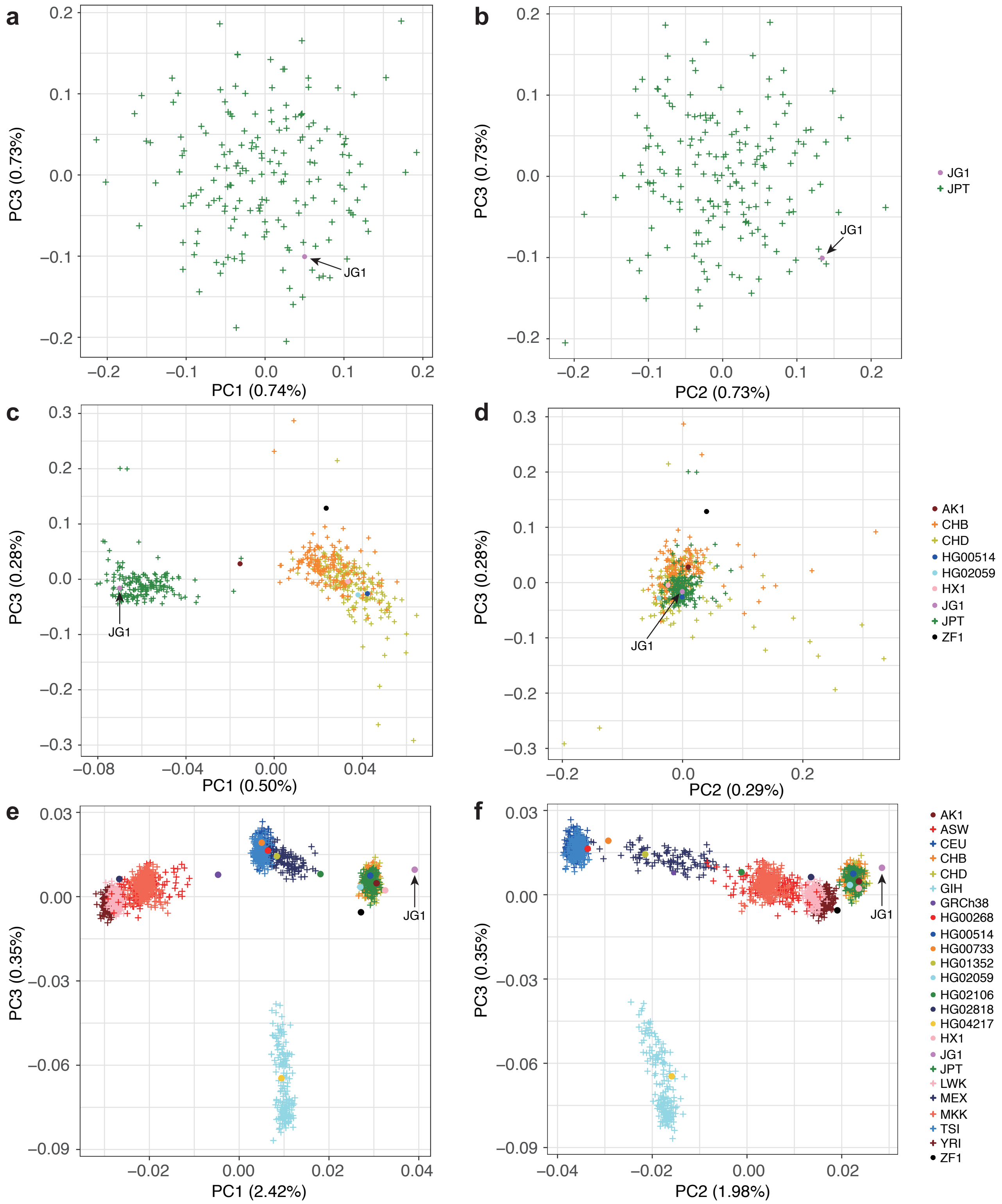

Supplementary Fig. 10: PCA plots with PC1 versus PC3 (**a**, **c**, **e**) or PC2 versus PC3 (**b**, **d**, **f**) for the JPT (**a**, **b**), the Asian (**c**, **d**), and world-wide haplotypes (**e**, **f**). The percentages in the X- and Y-axes indicate the variance explained by the principal component. Populations for the assemblies are provided in Supplementary Table 6. Source data are provided as a Source Data file.

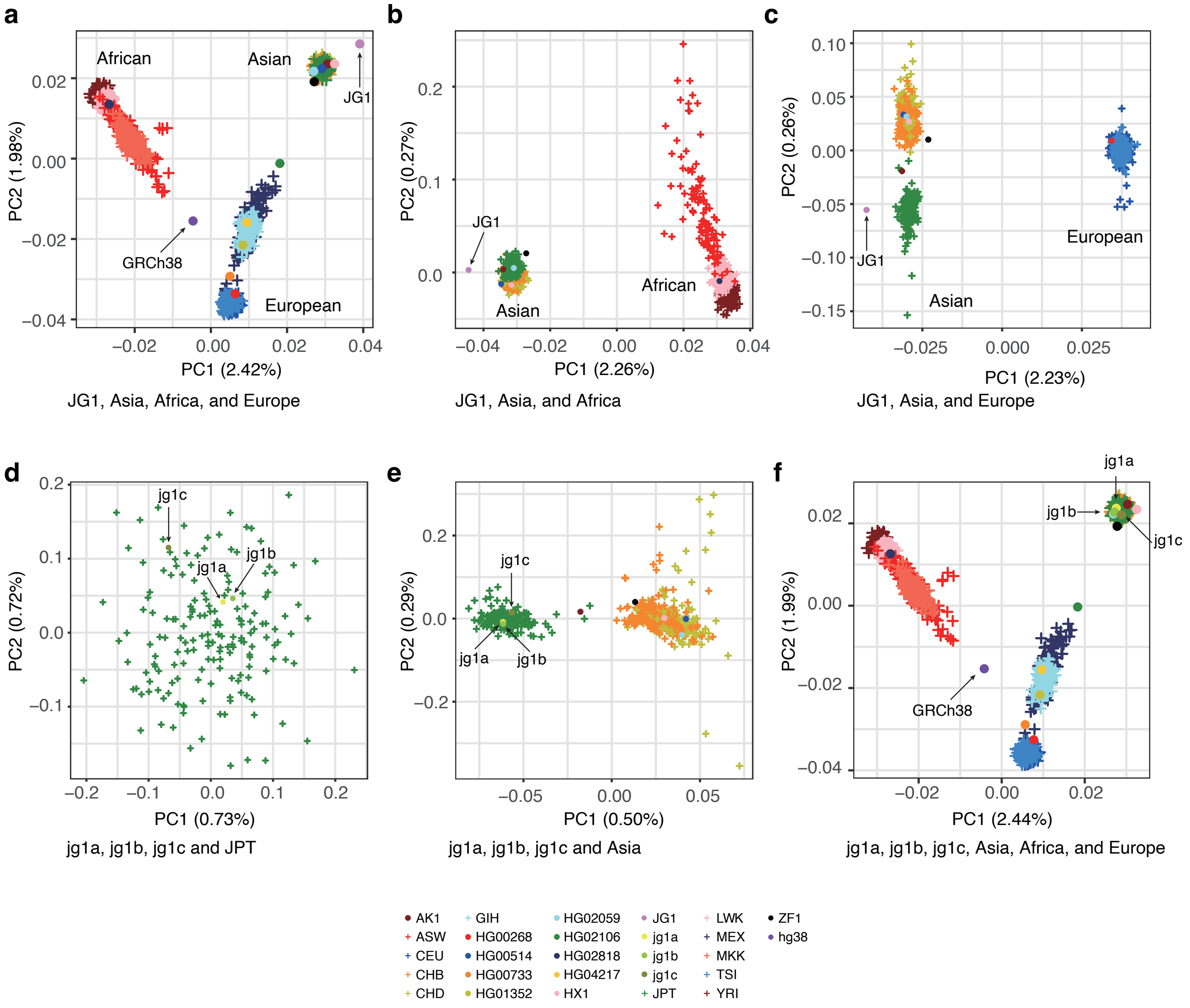

Supplementary Figure 11. PCA plots with world-wide populations. **a** PCA plot of the haplotype SNP composition of JG1, the reference GRCh38, 11 other high-quality assemblies, and world-wide HapMap3 samples. **b** PCA plot of JG1, Asian and African assemblies or HapMap3 samples. **c** PCA plot of JG1, Asian and European assemblies or HapMap3 samples. **d** PCA plot of the three base assemblies jg1a, jg1b, and jg1c and JPT HapMap3 samples. **e** PCA plot of jg1a, jg1b, and jg1c and Asian assemblies or HapMap3 samples. **f** PCA plot of jg1a, jg1b, and jg1c and world-wide assemblies or HapMap3 samples. PCA indicates principal component analysis. SNP indicates single nucleotide polymorphism. Populations for the assemblies are provided in Supplementary Table 6. Source data are provided as a Source Data file.

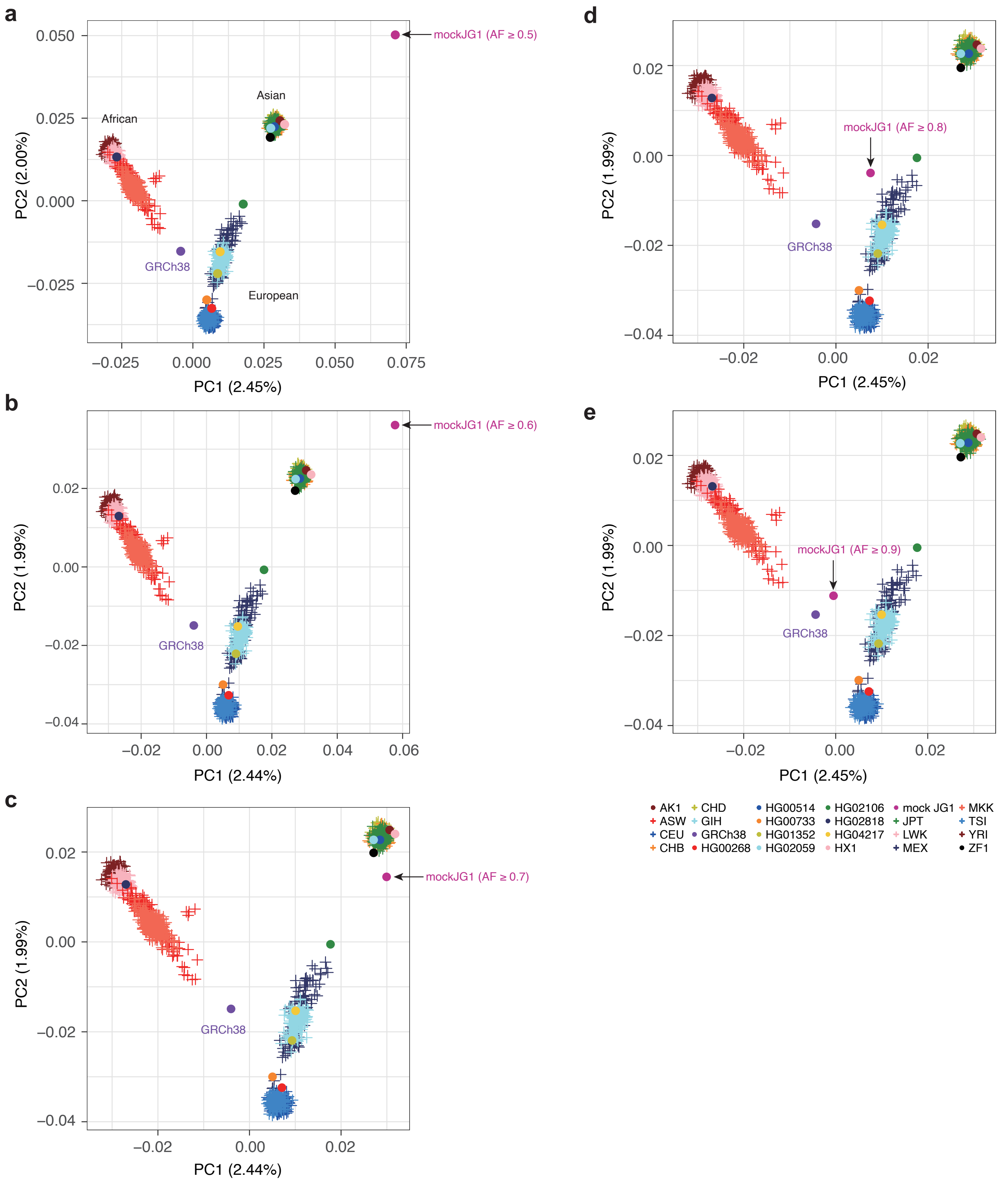

Supplementary Figure 12. PCA plots of "mock JG1" genomes. Shown are mock JG1 and the world-wide assemblies or HapMap3 samples; mock JG1 genomes were constructed by replacing the reference allele in hs37d5 with the major allele in the Japanese population for sites with **a** AF ≥ 0.5, **b** AF ≥ 0.6, **c** AF ≥ 0.7, **d** AF ≥ 0.8, and **e** AF ≥ 0.9 inferred from the allele frequency panel 3.5KJPNv2. Populations for the assemblies are provided in Supplementary Table 6. AF indicates Allele Freuquency. Source data are provided as a Source Data file.

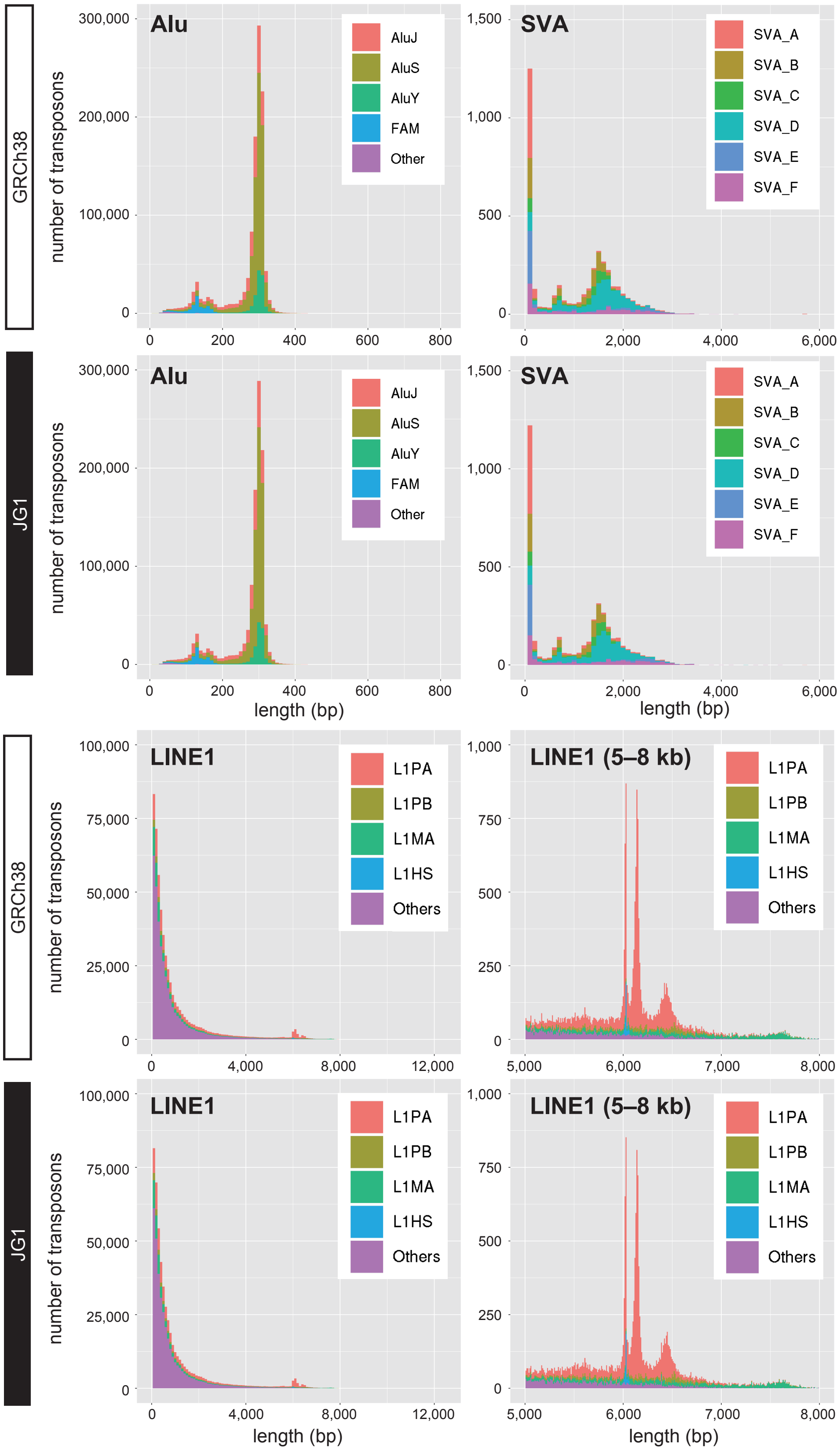

Supplementary Figure 13. Length distributions of detected transposable elements in the GRCh38 and JG1 genomes. Shown are *Alu*, SVA, and LINE1. Transposable elements and their subclasses were identified using RepeatMasker software (ver. 4.0.7) with the '-species human' option. The resulting OUT format files were converted to BED format using the rmsk2bed command of BEDOPS software[1] (ver. 2.4.35). Transposable elements disrupted by other elements were counted as distinct.

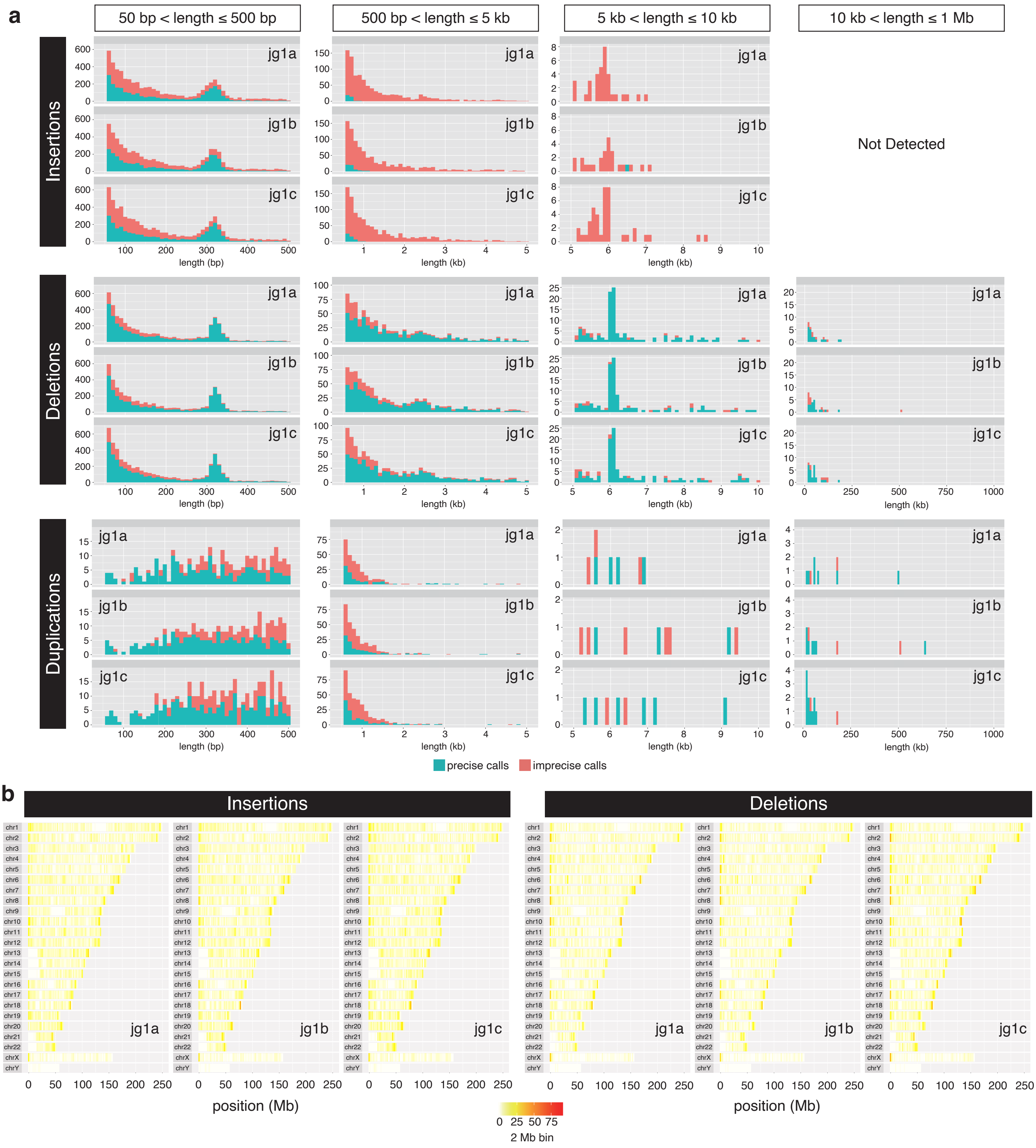

Supplementary Figure 14. Mapping-based SV analysis. Length histogram (**a**) and chromosomal distribution (**b**) of SVs (insertions, deletions, and duplications) detected by PacBio long-read mapping for the three individuals, jg1a, jg1b, and jg1c.

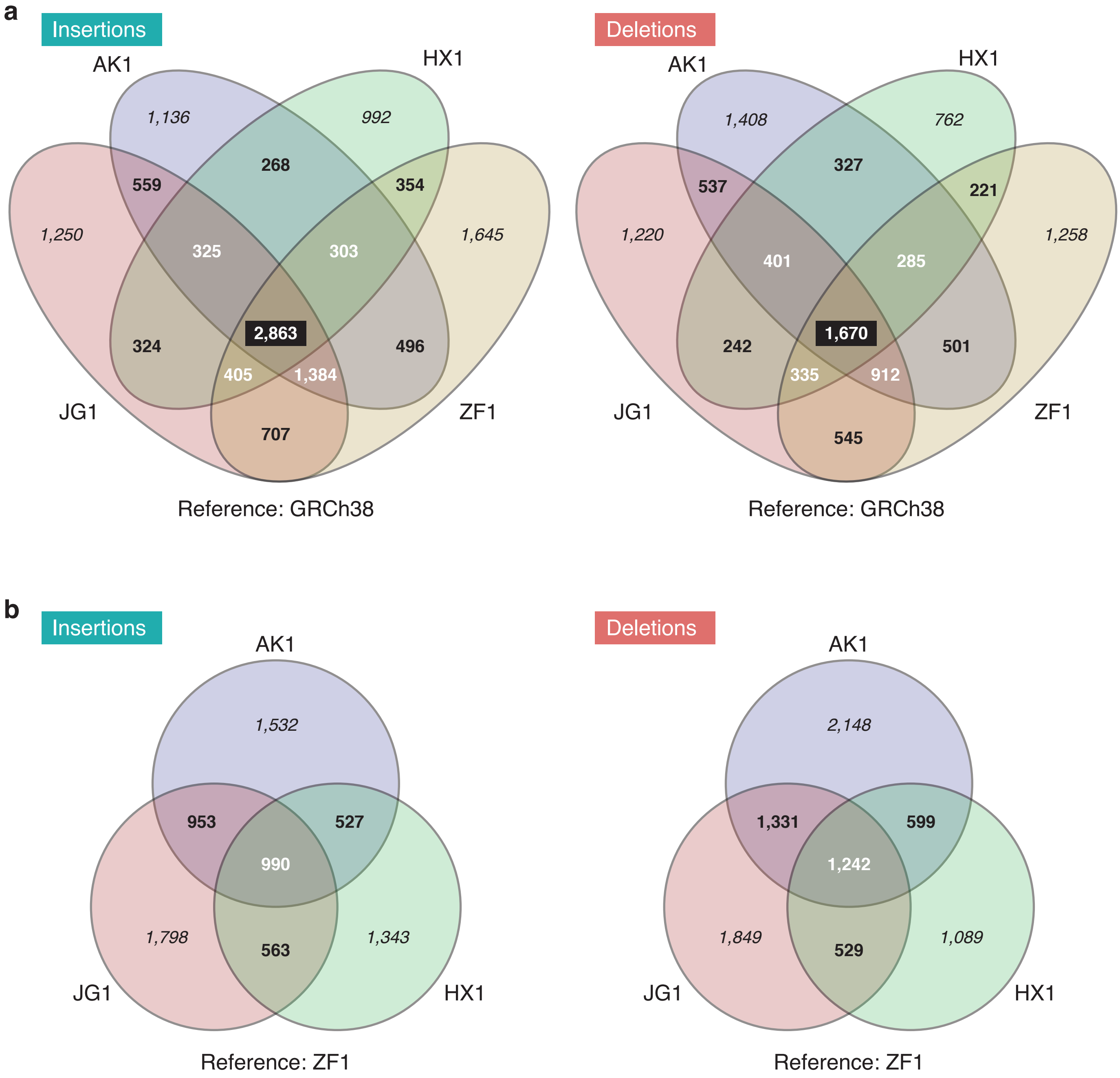

Supplementary Figure 15. Alignment-based SV analysis. **a** Venn diagram of insertions and deletions detected in JG1, AK1, HX1, and ZF1 by genome-by-genome alignment against the reference GRCh38. **b** Venn diagram of insertions and deletions detected in JG1, AK1, and HX1 by genome-by-genome alignment against ZF1. SV indicates Structural Variants.

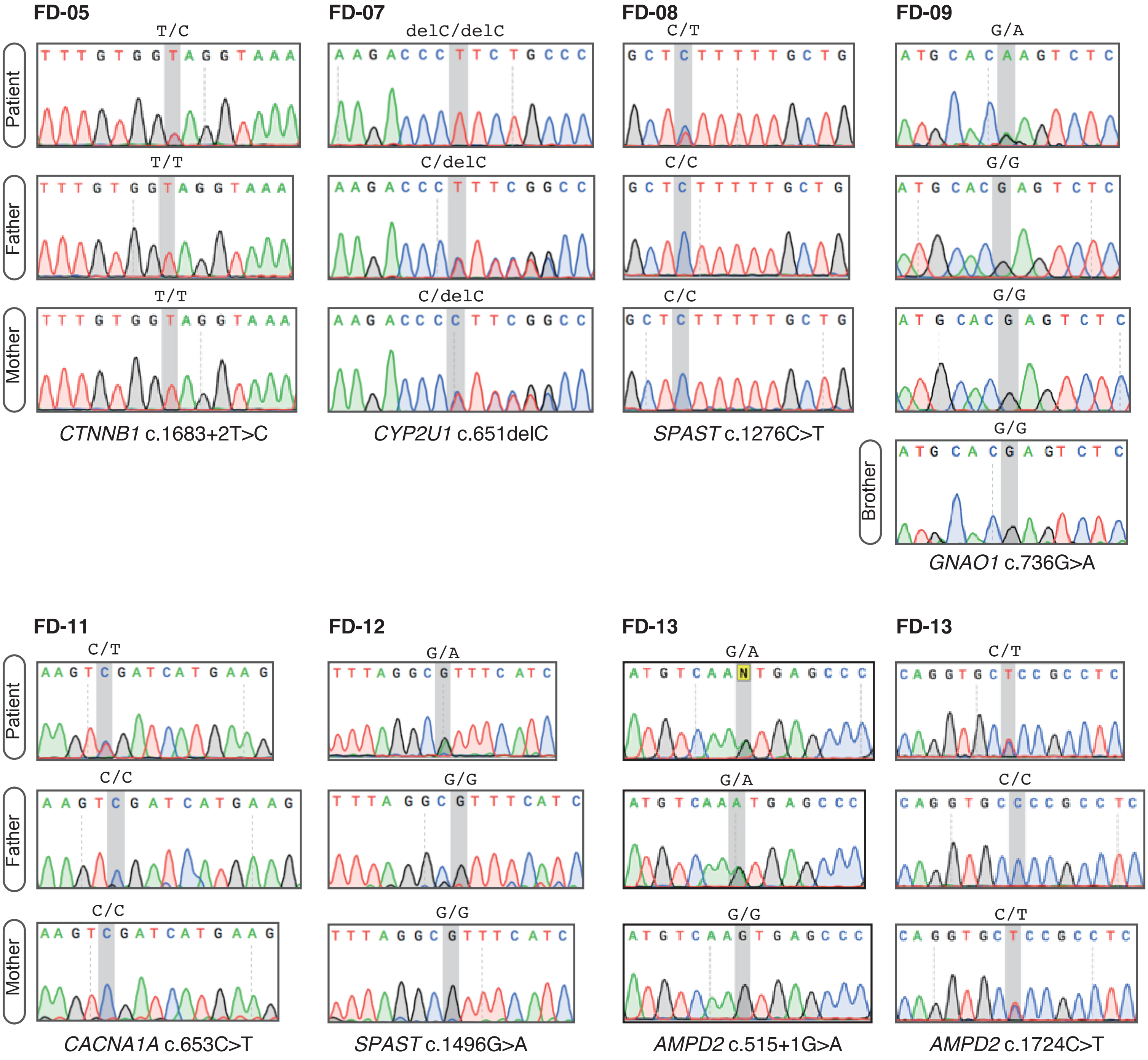

Supplementary Figure 16. Genomic Sanger sequencing validation of the variants successfully identified by exome analysis using JG1.

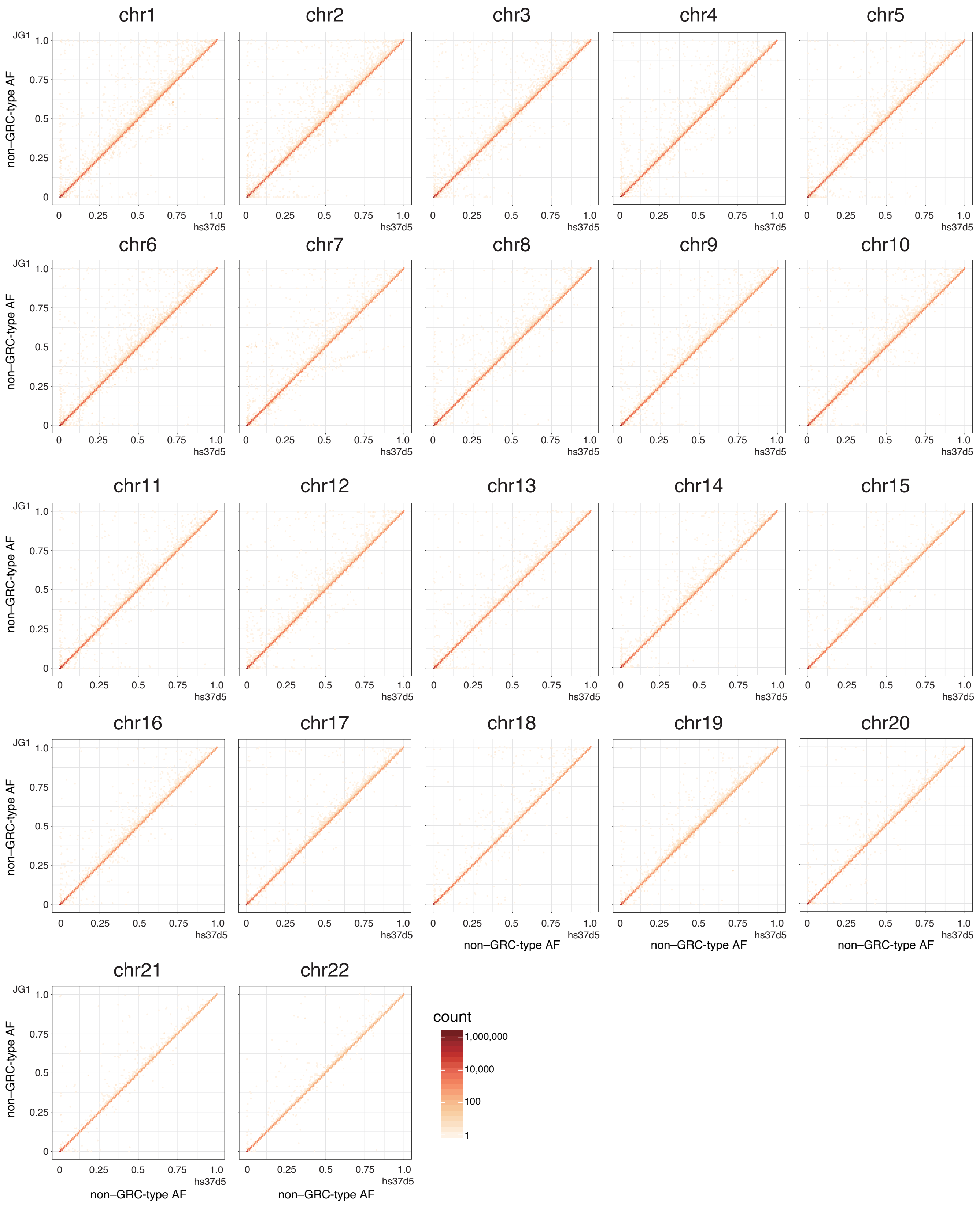

Supplementary Figure 17. Comparison of the allele frequency between JG1 and hs37d5. Shown are density plots comparing the allele frequency of the non–GRC-type variants detected by mapping whole-genome sequencing reads from 1,070 Japanese individuals to hs37d5 (X-axis) and JG1 (Y-axis).

Supplementary Table 1. Basic statistics of PacBio subreads.

Individual	Number of subreads	Sum of subread length (bp)	depth*
**jg1a**	34,445,474	364,777,563,591	122X
**jg1b**	36,798,731	370,437,373,175	123X
**jg1c**	41,535,337	383,220,406,482	128X

* Depth is calculated by assuming the genome size = 3.0 Gb.

Supplementary Table 2. Basic statistics of Bionano optical maps.

Individual	Enzyme	Number of optical maps	Sum of optical map length (bp)	depth*
**jg1a**	BspQI	1,156,682	368,075,072,000	123X
	BssSI	1,834,771	418,513,858,000	140X
**jg1b**	DLE-1	2,840,733	480,476,071,000	160X
**jg1c**	DLE-1	3,594,225	524,851,027,000	175X

* Depth is calculated by assuming the genome size = 3.0 Gb.

Supplementary Table 3. Basic statistics of Illumina paired-end and mate-pair reads.

Method	Individual	read length (bp)	# of reads	Sum of read length (bp)	depth*
**paired end**	jg1a	162	543,599,992	88,063,198,704	29X
		259	303,625,608	78,639,032,472	26X
	jg1b	162	578,161,124	93,662,102,088	31X
		259	319,177,020	82,666,848,180	28X
	jg1c	162	571,414,220	92,569,103,640	31X
		259	302,332,088	78,304,010,792	26X
**mate pair****	jg1a	201	189,189,310	38,027,051,310	13X
	jg1b		184,346,446	37,053,635,646	12X
	jg1c		185,928,504	37,371,629,304	12X

* Depth is calculated by assuming the genome size = 3.0 Gb.

**all reads (before library separation)

Supplementary Table 4. Assembly statistics for intermediate assemblies to construct JG1.

assembly	Total length (bp)	Contig		Scaffold		Number of misassemblies	Number of gap regions	N-gap length (bp)
		Number	N50 (bp)	Number	N50 (bp)			
jg1a primary contigs	2,855,392,439	2,194	20,631,146	NA	NA	1,912	0	0
jg1b primary contigs	2,852,624,381	2,227	21,603,629	NA	NA	1,673	0	0
jg1c primary contigs	2,851,554,649	2,120	19,616,169	NA	NA	1,673	0	0
jg1a scaffolds	2,889,327,167	NA	NA	1,911	86,280,884	2,071	417	34,337,088
jg1b scaffolds	2,880,572,022	NA	NA	1,893	59,380,744	1,762	413	28,327,001
jg1c scaffolds	2,875,657,275	NA	NA	1,797	58,198,703	1,867	380	24,482,557
meta-scaffolds* (jg1c + (jg1a + jg1b))	2,858,691,982	1,043	20,802,489	708	66,367,161	1,581	338	22,472,181

* reproduced from Table 1 for comparison.

NA: Not Applicable.

Supplementary Table 5. Basic statistics of Bionano assembly.

Individual	Enzyme	# of fragments	N50 (Mb)	Total length (Mb)
**jg1a**	BspQI	4,761	1.179	3846.912
**jg1a**	BssSI	4,392	1.034	3202.036
**jg1b**	DLE-1	581	41.761	3194.487
**jg1c**	DLE-1	496	64.293	3481.086

Supplementary Table 6. Comparison of basic assembly statistics with other high-quality assemblies.

	Population*	Sex**	Total length (bp)	Contig†		Scaffold††		Number of mis-assemblies	Numb-er of gaps	N-gap length (bp)	Reference or accession number
				Number	N50 (bp)	Number	N50 (bp)				
JG1	Japanese	M	3,085,782,898	1,068	23,601,496	624	141,953,703	1,654	473	251,127,232	this study
CHM1	Mole	-	3,037,883,181	40,852	143,936	24	155,181,468	639	40,916	210,229,881	GCF_000306695.2
CHM13	Mole	-	2,875,999,248	1,916	29,260,714	NA	NA	1,407	0	0	GCA_002884485.1
HG00268	FIN	F	2,908,574,082	1,995	20,033,786	NA	NA	2,226	0	0	GCA_008065235.1
HG00514	CHB	F	3,094,330,096	2,619	30,976,811	2,151	155,249,355	1,711	773	229,340,998	GCA_002180035.3
HG00733	PUR	F	2,882,001,880	566	22,162,569	NA	NA	1,788	0	0	GCA_002208065.1
HG01352	CLM	F	2,884,526,810	3,162	21,644,977	NA	NA	1,564	0	0	GCA_002209525.2
HG02059	KHV	F	2,898,275,003	3,180	25,293,151	NA	NA	2,081	0	0	GCA_003070785.1
HG02106	PEL	F	2,887,220,360	2,636	3,228,826	NA	NA	1,605	0	0	GCA_008583285.1
HG02818	GWD	F	2,875,770,361	3,267	22,499,404	NA	NA	1,757	0	0	GCA_003574075.1
HG04217	ITU	F	2,878,155,429	4,249	3,421,995	NA	NA	2,437	0	0	GCA_007821485.1
NA12878	CEU	F	3,088,210,890	2,996	17,089,889	2,142	155,131,045	1,599	1,124	236,915,269	GCA_002077035.3
NA19240	YRI	F	3,088,495,238	2,358	36,017,547	1,741	155,338,310	1,768	826	221,748,766	GCA_001524155.4
NA19434	LWK	F	2,864,262,081	3,123	21,513,396	NA	NA	1,449	1	1	GCA_002872155.1

* Mole: hydatidiform mole, population unknown; FIN: Finnish in Finland; CHB: Han Chinese in Beijing; PUR: Puerto Ricans from Puerto Rico; CLM: Columbians from Medellin, Colombia; KHV: Kinh in Ho Chi Minh City, Vietnam; PEL: Peruvians from Lima, Peru; GWD: Gambian in Western Divisions in the Gambia; ITU: Indian Telugu from the UK; CEU: Utah Residents (CEPH) with Northern and Western European Ancestry; YRI: Yoruba in Ibadan, Nigeria; LWK: Luhya in Webuye, Kenya.

** M: Male, F: Female

† Contigs statistics were assessed by breaking scaffolds at gap sites.

†† Scaffold statistics for JG1 was assessed on the pseudo-molecules.

NA: Not Applicable.

Supplementary Table 7. Base-error rate estimation

Sample	Length of covered region (bp)*	SNVs			Indels			Error rate	Unmapped reads
		homo	hetero	all	homo	hetero	all		
jg1a	2,828,472,312	4,043	2,524,105	2,528,148	24,793	760,068	784,861	1.02×10^{-5}	1.08%
jg1b	2,824,168,024	3,886	2,510,663	2,514,549	29,371	750,119	779,490	1.18×10^{-5}	0.66%
jg1c	2,825,737,567	4,159	2,522,881	2,527,040	37,106	748,041	785,147	1.46×10^{-5}	1.06%

* Covered region was defined as the region with the mapped read depth ranging from 5 to 149.

Supplementary Table 8. Length of consecutive Ns inserted manually.

chr	pter (bp)	cen (bp)	qter (bp)	References
**1**	10,000	30,000,000	10,000	2–4
**2**	10,000	3,000,000	10,000	
**3**	10,000	3,000,000	10,000	
**4**	10,000	3,000,000	10,000	
**5**	10,000	3,000,000	10,000	
**6**	10,000	3,000,000	10,000	
**7**	10,000	3,000,000	10,000	
**8**	10,000	-	10,000	
**9**	10,000	30,000,000	10,000	2–4
**10**	10,000	3,000,000	10,000	
**11**	10,000	-	10,000	
**12**	10,000	3,000,000	10,000	
**13**	16,000,000	-	10,000	2
**14**	16,000,000	-	10,000	2
**15**	17,000,000	-	10,000	2
**16**	10,000	20,000,000	10,000	2–4
**17**	10,000	3,000,000	10,000	
**18**	10,000	3,000,000	10,000	
**19**	10,000	3,000,000	10,000	
**20**	10,000	3,000,000	10,000	
**21**	11,000,000	-	10,000	
**22**	13,000,000	-	10,000	
**X**	10,000	3,000,000	10,000	
**Y**	2,260,577	3,000,000	30,000,000	2, 5–7

Supplementary Table 9. Assembly evaluation and comparison.

assembly	population	consensus quality vs GRCh38		Protein-truncating variants			gap filling			Reference or accession number
		covered region (%)	average identity (%)	SNVs	Indels	Total	uniquely filled gaps	filled gaps (total)	narrowed gaps (total)	
JG1	Japanese	**95.53**	**99.79**	**374**	**407**	**781**	**36**	**48**	267	this study
AK1	Korean	92.49	**99.78**	407	722	1,129	30	35	**295**	8
HX1	Chinese	91.17	99.64	403	10,330	10,733	27	34	**299**	9
ZF1	Tibetan	91.87	99.77	413	761	1,174	**34**	**52**	265	10
CHM1	mole	**95.80**	**99.80**	394	**415**	**809**	24	38	274	GCF_000306695.2
CHM13	mole	91.35	**99.78**	416	**406**	**822**	32	40	**282**	GCA_002884485.1
HG00268	FIN	91.78	**99.78**	**349**	1,842	2,191	31	39	257	GCA_008065235.1
HG00514	CHB	94.80	99.77	417	2,414	2,831	34	38	263	GCA_002180035.3
HG00733	PUR	91.55	99.77	**389**	1,881	2,270	30	33	256	GCA_002208065.1
HG01352	CLM	91.43	99.76	392	2,908	3,300	32	35	270	GCA_002209525.2
HG02059	KHV	91.67	99.77	392	1,920	2,312	**37**	41	267	GCA_003070785.1
HG02106	PEL	91.71	99.77	393	2,286	2,679	31	**48**	261	GCA_008583285.1
HG02818	GWD	91.50	99.74	446	2,411	2,857	33	35	258	GCA_003574075.1
HG04217	ITU	90.87	99.77	392	2,699	3,091	29	36	252	GCA_007821485.1
NA12878	CEU	95.22	99.77	410	2,319	2,729	32	36	271	GCA_002077035.3
NA19240	YRI	**95.27**	99.75	457	2,250	2,707	33	44	266	GCA_001524155.4
NA19434	LWK	91.20	99.74	439	2,116	2,555	30	33	278	GCA_002872155.1

The values for the top three assemblies were written in bold for each category.

Supplementary Table 10. Evaluation of AUGUSTUS gene prediction

		Number of genes	Number of multi-exon transcripts	Base level		Exon level		Intron level		Intron chain level		Transcript level		Locus level	
				Sensitivity	Precision	Sensitivity	Precision	Sensitivity	Precision	Sensitivity	Precision	Sensitivity	Precision	Sensitivity	Precision
JG1	AUGUSTUS	34,868	23,542	41.6%	64.3%	61.9%	53.2%	63.0%	57.7%	6.7%	5.2%	1.0%	0.5%	1.0%	0.5%
	GENCODE	18,105	18,528												
GRCh38	AUGUSTUS	30,329	23,965	41.9%	67.6%	62.0%	54.4%	63.0%	57.5%	6.7%	5.3%	1.1%	0.7%	1.2%	0.7%
	GENCODE	18,553	18,886												

Supplementary Table 11. Alignment-based SNV calls supported by independent short- or long-read mapping experiments

platform	variants	SNVs	
		number	% support
multi-platform	Alignment-based SNV calls	2,501,575	-
DNBseq	SNVs supported by ≥ 1 sample	2,349,454	93.9%
	SNVs supported by ≥ 2 samples	2,325,341	93.0%
	SNVs supported by 3 samples	1,751,325	70.0%
Nanopore	SNVs supported by ≥ 1 sample	2,399,537	95.9%
	SNVs supported by ≥ 2 samples	2,369,201	94.7%
	SNVs supported by 3 samples	1,782,656	71.3%

Supplementary Table 12. Basic statistics of DNBseq reads.

Individual	read length (bp)	Number of reads	Sum of read length (bp)	depth*
**jg1a**	150	1,023,216,250	153,482,437,500	51X
**jg1b**	150	925,140,642	138,771,096,300	46X
**jg1c**	150	849,670,416	127,450,562,400	42X

* Depth is calculated by assuming the genome size = 3.0 Gb.

Supplementary Table 13. Basic statistics of nanopore long reads.

protocol	Individual	Number of reads	Sum of read length (bp)	depth*
**super-long**	jg1a	4,541,246	102,914,186,094	34X
	jg1b	5,822,163	129,786,104,302	43X
	jg1c	5,285,674	97,029,929,831	32X
**long**	jg1a	5,616,707	35,691,417,262	12X
	jg1b	4,189,603	26,123,895,368	9X
	jg1c	4,225,469	26,808,029,958	9X

* Depth is calculated by assuming the genome size = 3.0 Gb.

** Shown are statistics after filtering (Phred-scaled read quality > 6 and 100-bp head- and tail-cropping).

Supplementary Table 14. SV calls based on PacBio long-read mapping by NGMLR and SV calling by Sniffles

Individual	Insertions	Deletions	Duplications	Inversions	Translocations	Others	Total
**jg1a**	7,101	6,164	629	61	37	8	14,000
**jg1b**	7,059	5,905	702	85	48	10	13,809
**jg1c**	7,679	6,718	815	84	47	5	15,348

* Translocations indicate those labeled as BND (breakends) by Sniffles software.

** Others include inverted duplications (INVDUP), ambiguous SV calls between duplications and insertions (DUP/INS).

Supplementary Table 15. SV calls based on Nanopore super-long read mapping by minimap2 and SV calling by Sniffles

Individual	Insertions	Deletions	Duplications	Inversions	Translocations	Others	Total
**jg1a**	10,785	8,007	67	65	169	6	19,099
**jg1b**	11,479	8,605	93	91	209	7	20,484
**jg1c**	10,232	7,817	64	60	133	7	18,313

* Translocations indicate those labeled as BND (breakends) by Sniffles software.

** Others include inverted duplications (INVDUP), ambiguous SV calls between duplications and insertions (DUP/INS) or deletions and inversions (DEL/INV).

Supplementary Table 16. Alignment-based SV calls supported by orthogonal or independent mapping experiments

**platform**	**variants**	**Insertions**		**Deletions**	
		**number**	**% support**	**number**	**% support**
multi-platform	Alignment-based SV calls*	7,825	-	5,875	-
PacBio	SVs supported by ≥ 1 sample	5,856	74.8%	4,779	81.3%
	SVs supported by ≥ 2 samples	4,243	54.2%	3,758	64.0%
	SVs supported by 3 samples	2,430	31.1%	2,059	35.0%
Nanopore	SVs supported by ≥ 1 sample	7,060	90.2%	5,103	86.9%
	SVs supported by ≥ 2 samples	5,401	69.0%	4,221	71.8%
	SVs supported by 3 samples	3,242	41.4%	2,524	43.0%

* The raw number of alignment-based SV calls were 8,697 insertions and 6,190 deletions, in which some variant calls were regarded identical by SURVIVOR merge software, and hence the number was reduced to 90–95%.

Supplementary Table 17. Sanger sequencing validation of exome-identified variants.

variants	JG1	hs37d5
Total number of Sanger-detected variants / exome-detected variants*	57/58	57/58
SNVs	52/53	52/53
Indels	4/4	4/4
Total number of exome-detected variants / Sanger-detected variants**	57/59	57/59
SNVs	53/54	53/54
Indels	4/5	4/5
Total number of Ref/Alt switched variants between JG1 and hs37d5	7	
Ref allele of JG1 was major in 3.5KJPNv2	5/7	
SNVs		4/5
Indels		1/5
Ref allele of JG1 was minor in 3.5KJPNv2	2/7	
SNVs		2/2
Indels		0/2

* The 1/58 false-positive variant, which was detected by exome analysis but not by Sanger sequencing was the same between JG1 and hs37d5.

** The 2/59 false-negative variants, which were detected by Sanger sequencing but not by exome analysis were the same between JG1 and hs37d5.

Supplementary Table 18. Effect of genic indel-specific correction of individual assembly.

subject/ assembly	indel correction	protein-truncating variants		
		SNVs	indels	total
jg1a	before	418	561	979
	after	441	313	754
jg1b	before	425	528	953
	after	434	359	793
jg1c	before	408	566	974
	after	439	374	813
JG1	-	374	407	781

Indel correction pipeline described in Kronenberg et al.[11] was applied to each individual assembly. Variants were called through genome-by-genome alignment against GRCh38 by minimap2 and paftools software call command[12]. Protein-truncating variants were identified by SnpEff software[13].

Supplementary Methods: Manual modification of consecutive N-gap regions.

The physical lengths of the short arms of acrocentric chromosomes 13, 14, 15, 21, and 22 were obtained from Table 4 of Morton (1991)[2]. The relative length estimates of constitutive heterochromatin regions in the chromosome 1, 9, 16 were obtained from ref. 3 and ref. 4. The relative length estimate of heterochromatin segment of the Y chromosome was obtained from refs. 5–7. These relative lengths were converted to the base-pair length (Mb) by using the chromosomal arms shown in Table 4 of Morton (1991)[2]. The length of consecutive Ns for each chromosome is provided in Supplementary Table 8. For all chromosomes except 8 and 11, 3-Mb consecutive Ns were inserted instead of 10-kb Ns inserted by the ALLMAPS software, between the two scaffolds flanking the centromere. For chromosomes 8 and 11, in which the centromere-specific sequence repeats were identified in the midst of a scaffold by aligning the LinearCen1.1 sequences[14] using minimap2 software, no centromeric Ns were inserted. The position of the centromere was inferred from the Whitehead-RH and GeneMap99-GB4 maps, in which the centromeric or constitutive heterochromatin region could be inferred from the region sparsely covered by STS markers, possibly due to the radiation conditions.

Supplementary Note 1: Majority decision and erroneous base inclusion.

We aligned each individual draft assembly (short-read polished hybrid assembly) to the meta-scaffold (jg1c + (jg1a + jg1c)) using minimap2, and variants were called using the paftools software call command, resulting in VCF files. The total number of multi-allelic sites was 120,937 (1,139 SNV–SNV sites, 11,220 SNV–indel sites, and 108,578 indel–indel sites). After randomly choosing one allele as the reference, 4,514 SNV alleles and 75,894 indel alleles (80,408 total variants, corresponding to 66.5% of all multi-allelic sites) were adopted as the new reference alleles. By inspecting the INFO field of the VCF file, we could extract the position of the newly incorporated allele on the coordinate of each individual assembly. We then examined whether the position was labeled as erroneous by the procedure of the base-error rate estimation and found that 4 SNVs and 16 indels were labeled as erroneous. Because the erroneous sites were inspected for two assemblies per each site, the total number of estimated erroneous bases would be 4 × (3 ÷ 2) SNVs + 16 × (3 ÷ 2) indels = 6 SNVs + 24 indels = 30 sites. Although this majority decision strategy could not prevent all erroneous bases from being chosen, the number of such sites was just 30 per 2.86 Gb (0.000001%), or 0.025% of all multi-allelic sites, which we deemed acceptable.

Supplementary Note 2. Evaluation of *de novo* gene prediction.

We performed *de novo* gene predictions by AUGUSTUS software for the main chromosomes of GRCh38 (chromosomes 1–22, X, Y, and M) and compared the results with the GENCODE ver. 29 dataset (Supplementary Fig. 9). We considered the longest transcript per protein-coding gene from GENCODE because AUGUSTUS outputs one transcript per one gene. AUGUSTUS predicted more genes than the GENCODE dataset, primarily because AUGUSTUS predicted the larger number of short transcripts. Comparing the two datasets demonstrated that AUGUSTUS predict the larger number of gene regions with exon-level sensitivity higher than 60% (Supplementary Table 10). We observed similar results for JG1. These results suggested that AUGUSTUS can predict candidate novel genes with a modest, if not very high, sensitivity. However, no protein-coding genes were predicted for the 36 putative novel sequences that filled the GRCh38 gaps. Therefore, these 36 regions either do not encode any protein-coding genes or the gene prediction software is still not sensitive enough to detect possible genes.

Supplementary Note 3. PCA of JG1 haplotype

Why was JG1 plotted outside the Asian cluster? The plotted location of JG1 was indeed outside the Asian cluster, but more importantly, the position was in a more distant site both from the European and African clusters. This pattern means that JG1 was more distant from European and African populations than any haplotype in the Asian population, i.e., the genetic composition of JG1 was more distant from the two populations. This was confirmed by the fact that JG1 was plotted at the most distant site from the European or African populations when we performed PCA using Asian and African (Supplementary Fig. 11b) or Asian and European populations (Supplementary Fig. 11c), respectively. On the other hand, the base assemblies jg1a, jg1b, and jg1c, which were used to construct JG1, were plotted in the Japanese population (Supplementary Fig. 11d–f).

Then, why was JG1 located away from both European and African populations? JG1 adopted the major allele among the three assemblies jg1a, jg1b, and jg1c in its construction process. This majority decision resulted in adopting the major allele in the Japanese population at most SNP sites (Fig. 2c). The major allele in a Japanese population is often minor in European or African populations and vice versa. Therefore, JG1 can be considered more non-European and non-African than any Japanese haplotype or even any Asian haplotype. For these reasons, JG1 was located distant from both European and African clusters in the PCA.

To gain further insights into the effect of this adoption of the major allele on PCA plot location, we generated "mock JG1" haplotypes that replaced the reference genome hs37d5 with the major allele (non-reference allele frequency 50%–90%) in the Japanese population and performed PCA (Supplementary Fig. 12). We found that replacing the GRC-type allele with an allele harbored by ≥ 50% or 60% of the Japanese haplotypes plotted outside the Asian cluster and distant from European and African populations just like JG1 (Supplementary Fig. 12).

SUPPLEMENTARY REFERENCES

1. Neph, S. *et al.* BEDOPS: high-performance genomic feature operations. *Bioinformatics* **28**, 1919-1920 (2012).
2. Morton, N. E. Parameters of the human genome. *Proc. Natl. Acad. Sci. USA* **88**, 7474-7476 (1991).
3. Ludgren, R. *et al.* Constitutive heterochromatin C-band polymorphism in prostatic cancer. *Cancer Genet. Cytogenet.* **51**, 57-62 (1991).
4. Podugolnikova, O. A. & Blumina, M. G. Heterochromatic regions on chromosomes 1, 9, 16, and Y in children with some disturbances occurring during embryo development. *Hum. Genet.* **63**, 183-188 (1983).
5. Petković, I. *et al.* Heterochromatic segment length of Y chromosome in 55 boys with malignant diseases. *Cancer Genet. Cytogenet.* **25**, 351-353 (1987).
6. Erçal, M. D. & Bröndum-Nielsen, K. Length polymorphism of heterochromatic segment of the Y chromosome in boys with acute leukemia. *Acta Paediatr. Jpn.* **37**, 614-616 (1995).
7. Petković, I. Variability of euchromatic and heterochromatic segment of the Y chromosome in men with malignant tumors and in a control group. *Cancer Genet. Cytogenet.* **13**, 29-36 (1984).
8. Seo, J.-S. *et al.* *De novo* assembly and phasing of a Korean human genome. *Nature* **538**, 243-247 (2016).
9. Shi, L. *et al.* Long-read sequencing and *de novo* assembly of a Chinese genome. *Nat. Commun.* **7**, 12065 (2016).
10. Ouzhuluobu *et al.* *De novo* assembly of a Tibetan genome and identification of novel structural variants associated with high altitude adaptation. *Natl. Sci. Rev.* **7**, 391-402 (2020).
11. Kronenberg, Z. N. *et al.* High-resolution comparative analysis of great ape genomes. *Science* **360**, eaar6343 (2018).
12. Li, H. Minimap2: pairwise alignment for nucleotide sequences. *Bioinformatics* **34**, 3094-3100 (2018).
13. Cingolani, P. *et al.* A program for annotating and predicting the effects of single nucleotide polymorphisms, SnpEff: SNPs in the genome of *Drosophila melanogaster* strain w^{1118}*; iso-2; iso-3. Fly (Austin)* **6**, 80-92 (2012).
14. Miga, K. H. *et al.* Centromere reference models for human chromosomes X and Y satellite arrays. *Genome Res.* **24**, 697-707 (2014).
