# Supplementary material for: Construction and integration of three de novo Japanese human genome assemblies toward a population-specific reference
Source: Nat Commun. 2021 Jan 11;12:226. doi: 10.1038/s41467-020-20146-8 (PMC7801658; doi:10.1038/s41467-020-20146-8)
Supplement: Supplementary file 3 — Reporting Summary [file 41467_2020_20146_MOESM3_ESM.pdf]

nature research

Corresponding author(s): Kengo Kinoshita, Masayuki Yamamoto, Gen Tamiya

Last updated by author(s): Oct 29, 2020

Reporting Summary

Nature Research wishes to improve the reproducibility of the work that we publish. This form provides structure for consistency and transparency in reporting. For further information on Nature Research policies, see Authors & Referees and the Editorial Policy Checklist .

Statistics

For all statistical analyses, confirm that the following items are present in the figure legend, table legend, main text, or Methods section.

n/a	Confirmed	
☐	☒	The exact sample size (*n*) for each experimental group/condition, given as a discrete number and unit of measurement
☐	☒	A statement on whether measurements were taken from distinct samples or whether the same sample was measured repeatedly
☐	☒	The statistical test(s) used AND whether they are one- or two-sided<br>*Only common tests should be described solely by name; describe more complex techniques in the Methods section.*
☒	☐	A description of all covariates tested
☒	☐	A description of any assumptions or corrections, such as tests of normality and adjustment for multiple comparisons
☐	☒	A full description of the statistical parameters including central tendency (e.g. means) or other basic estimates (e.g. regression coefficient) AND variation (e.g. standard deviation) or associated estimates of uncertainty (e.g. confidence intervals)
☐	☒	For null hypothesis testing, the test statistic (e.g. *F*, *t*, *r*) with confidence intervals, effect sizes, degrees of freedom and *P* value noted<br>*Give P values as exact values whenever suitable.*
☒	☐	For Bayesian analysis, information on the choice of priors and Markov chain Monte Carlo settings
☒	☐	For hierarchical and complex designs, identification of the appropriate level for tests and full reporting of outcomes
☒	☐	Estimates of effect sizes (e.g. Cohen's *d*, Pearson's *r*), indicating how they were calculated

Our web collection on statistics for biologists contains articles on many of the points above.

Software and code

Policy information about availability of computer code

Data collection	SMRT Analysis ver. 2.2.0; Irys ICS ver. 1.5.4; Saphyr ICS ver. 3.1.4.3; bcl2fastq ver. 1.8.4; Guppy ver. 3.2.4
Data analysis	EIGENSOFT ver. 4.2; plink ver. 1.9; Falcon ver. falcon-2017.11.02-16.04-py2.7-ucs2.tar.gz; ArrowGrid ver. 81b03f1; BionanoSolve ver. 3.1 and 3.2; BWA MEM ver. 0.7.17; Picard tools ver. 2.18.4; SAMtools ver. 1.8; Pilon ver. 1.22; Metassembler ver. 1.5; MUMmer ver. 4.0.0beta2; NxTrim ver. 0.4.3; Bowtie2 ver. 2.3.4.1; minimap2 ver. 2.12 and 2.17; BCFtools ver 1.8 and 1.9; gPCR ver. 2.6a; ALLMAPS ver. 0.8.12; BEDTools ver. 2.26.0 and 2.27.1; NCBI Genome Decoration Page (https://www.ncbi.nlm.nih.gov/genome/tools/gdp); seqtk ver. 1.3; minidot ver. 0.2; GATK ver. 3.7.0, 4.0, 4.1, and 4.1.2.0, ; SnpEff ver. 4.3T; R ver. 3.5.1; ggplot2 ver. 3.0.0; mosdepth ver. 0.2.8; uniline ver. 99969cc; LiftMap.py (https://genome.sph.umich.edu.wiki.LiftMap.py); Clair ver. 2.0.7; SURVIVOR ver. 1.0.6; NGMLR ver. 0.2.7; Sniffles; ver 1.0.11; BLAST ver. 2.10.0+; CrossMap ver. 0.3.4; TraceTuner (sourceforge.net/projects.tracetuner/); Circa ver. 1.2.1; RepeatMasker ver. 4.0.7; BEDOPS ver. 2.4.35; Quast-LG ver. 5.0.0; AUGUSTUS ver. 3.3; gffcompare v0.11.6<br>Custom scripts are available from the Github repository (https://github.com/juntkym/JG1-paper)

For manuscripts utilizing custom algorithms or software that are central to the research but not yet described in published literature, software must be made available to editors/reviewers. We strongly encourage code deposition in a community repository (e.g. GitHub). See the Nature Research guidelines for submitting code & software for further information.

Data

Policy information about availability of data

All manuscripts must include a data availability statement. This statement should provide the following information, where applicable:
- Accession codes, unique identifiers, or web links for publicly available datasets
- A list of figures that have associated raw data
- A description of any restrictions on data availability

JG1 sequence, chain files and GENCODE annotation files are available from the jMorp website (https://jmorp.megabank.tohoku.ac.jp/201911/downloads#sequence).

DDBJ accession numbers for the 624 pseudo-molecules/scaffold sequences of JG1 are AP023461¬–AP024084 (https://getentry.ddbj.nig.ac.jp/getentry/na/).
BioProject and BioSample accession numbers are PRJDB10452 and SAMD00243993, respectively.
Individual sequencing and optical mapping datasets were deposited to National Bioscience Database Center (NBDC) Human Database under accession number hum0248.
Other datasets are available from Zenodo repository.
Raw data for the following figures are available as a Source Data: Figure 1a, 2a, 2b, 3d, 4a, 4b, Supplementary Fig. 10a–f, 11a–f, 12a–e.
SnpEff GRCh38.86 database can be downloaded by the SnpEff download command as "java -jar SnpEff.jar download GRCh38.86".
Blast nt database: ftp://ftp.ncbi.nlm.nih.gov/blast/db
UniSTS database: ftp://ftp.ncbi.nih.gov/pub/ProbeDB/legacy_unists/
GENCODE database: https://www.gencodegenes.org/human/release_29.html.

Field-specific reporting

Please select the one below that is the best fit for your research. If you are not sure, read the appropriate sections before making your selection.

☒ Life sciences ☐ Behavioural & social sciences ☐ Ecological, evolutionary & environmental sciences

For a reference copy of the document with all sections, see nature.com/documents/nr-reporting-summary-flat.pdf

Life sciences study design

All studies must disclose on these points even when the disclosure is negative.

Sample size	(1) For de novo genome assemblies, three subjects were chosen; No sample size calculation was performed; however, after assembly, we assessed whether they represent the genetic variation of the Japanese population in the manuscript.<br>(2) PCA for the three subjects was performed with 310 samples from the 3.5KJPNv2 allele frequency panel so that the 310 samples represent northern, western, and southern regions in Japan. No sample size calculation was performed.<br>(3) For the average depth analysis among the SV regions, we used 100 male and 100 female individuals with the earliest ID number in the 3.5KJPNv2 allele frequency panel; 200 samples are empirically determined to be considered sufficient to infer the average short-read mapping depth.<br>(4) For the rare-disease exome re-analysis, no sample size calculation was performed; however, we used the seven families who were analyzed using the same exome capture kits (SureSelect V5) in the nine families with successful causal variant identification described in Takezawa et al. (2018).<br>(5) For the exome PPV and Recall evaluations, we used additional 24 families with their genomic region(s) harboring their candidate variants analyzed by Sanger sequencing. No sample size calculation was performed, but this sample size was the result of collecting as much available Sanger sequence data as possible.<br>(6) For the applicability of WGS to JG1, no sample size calculation was performed, but we used all (1,070) individuals used in Nagasaki et al. (2015).
Data exclusions	For PCA of high-quality assemblies of samples described in Audano et al. (2019), NA12878, NA19240, and NA19434 were excluded because haplotypes of their close relatives were included in the HapMap3 samples (Supplementary Fig. 10–12). For PCA of the JG1 haplotype with Asian samples (Fig. 2b), four CHD samples were excluded because they suggested apparent inconsistency with the other Asian-origin samples in a PCA plot that includes them. For PCA of JG1, Asian, and African or European populations (Supplementary Fig. 11b, c), NA18138 CHD haploids were also excluded because they suggested apparent inconsistency with the other Asian-origin samples. These exclusion criteria were determined after observing the PCA plots that include them. The exclusion of related (including self) individuals is predetermined because the inclusion of such individuals is known to deform the inference of population structure substantially. Excluding samples with the apparent inconsistency of population ancestry, while somewhat arbitrary, is essential for visually inspecting the population structure among the samples of interest.
Replication	The objective is to construct a single set of haploid genome sequences that reflect the genetic diversity of the Japanese population, and thus the resulting genome sequence itself is not subject to replicability. The fact that the assembly statistics were not substantially different among the three demonstrate that the assembly workflow is replicable.
Randomization	Randomization is not applicable because this study is not designed to test the effects of interventions in sample individuals.
Blinding	Blinding is not applicable because this study is not designed to test the effects of interventions in sample individuals.

Reporting for specific materials, systems and methods

We require information from authors about some types of materials, experimental systems and methods used in many studies. Here, indicate whether each material, system or method listed is relevant to your study. If you are not sure if a list item applies to your research, read the appropriate section before selecting a response.

Materials & experimental systems

n/a	Involved in the study
☒	☐ Antibodies
☒	☐ Eukaryotic cell lines
☒	☐ Palaeontology
☒	☐ Animals and other organisms
☐	☒ Human research participants
☒	☐ Clinical data

Methods

n/a	Involved in the study
☒	☐ ChIP-seq
☒	☐ Flow cytometry
☒	☐ MRI-based neuroimaging

Human research participants

Policy information about studies involving human research participants

Population characteristics	All three JG1 subjects are males, which was confirmed by G-banding analysis, and have a self-reported Japanese origin, which was assessed by PCA. They are self-reportedly healthy, and their age was 45–59 years old.
Recruitment	Three male Japanese volunteers were recruited and participated in this study with written, informed consent. There may be a potential self-selection bias to select those confident to be healthy. This self-selection bias is expected to have little effect on selecting reference allele because of the majority-decision strategy.
Ethics oversight	This study was approved by the Research Ethical Committee of Tohoku Medical Megabank Organization and the Ethics Committee of Tohoku University Graduate School of Medicine.

Note that full information on the approval of the study protocol must also be provided in the manuscript.
