# Supplementary material for: Construction and integration of three de novo Japanese human genome assemblies toward a population-specific reference
Source: Nat Commun. 2021 Jan 11;12:226. doi: 10.1038/s41467-020-20146-8 (PMC7801658; doi:10.1038/s41467-020-20146-8)
Supplement: Supplementary file 4 — Description of Additional Supplementary Files [file 41467_2020_20146_MOESM4_ESM.pdf]

Description of Additional Supplementary Files

Supplementary Data 1
Non-reference sequences found in JG1

Supplementary Data 2
Non-reference sequences found in JG1 but not in Kher et al. 2017 or Wong et al. 2018

Supplementary Software.zip
The minimap2-to-liftover-chain.py script to create chain files from minimap2 output, and
The liftover-gencode2.py script to lift over Gencode annotation
